# Predicting daily recovery during long-term endurance training using machine learning analysis

**DOI:** 10.1007/s00421-024-05530-2

**Published:** 2024-06-20

**Authors:** Jeffrey A. Rothschild, Tom Stewart, Andrew E. Kilding, Daniel J. Plews

**Affiliations:** 1 Sports Performance Research Institute New Zealand (SPRINZ), Auckland University of Technology, Auckland, New Zealand; 2 High Performance Sport New Zealand, Auckland, New Zealand

**Keywords:** Training load monitoring, Cycling, Running, Triathlon, Nutrition, Sleep, HRV

## Abstract

**Purpose:**

The aim of this study was to determine if machine learning models could predict the perceived morning recovery status (AM PRS) and daily change in heart rate variability (HRV change) of endurance athletes based on training, dietary intake, sleep, HRV, and subjective well-being measures.

**Methods:**

Self-selected nutrition intake, exercise training, sleep habits, HRV, and subjective well-being of 43 endurance athletes ranging from professional to recreationally trained were monitored daily for 12 weeks (3572 days of tracking). Global and individualized models were constructed using machine learning techniques, with the single best algorithm chosen for each model. The model performance was compared with a baseline intercept-only model.

**Results:**

Prediction error (root mean square error [RMSE]) was lower than baseline for the group models (11.8 vs. 14.1 and 0.22 vs. 0.29 for AM PRS and HRV change, respectively). At the individual level, prediction accuracy outperformed the baseline model but varied greatly across participants (RMSE range 5.5–23.6 and 0.05–0.44 for AM PRS and HRV change, respectively).

**Conclusion:**

At the group level, daily recovery measures can be predicted based on commonly measured variables, with a small subset of variables providing most of the predictive power. However, at the individual level, the key variables may vary, and additional data may be needed to improve the prediction accuracy.

**Supplementary Information:**

The online version contains supplementary material available at 10.1007/s00421-024-05530-2.

## Introduction

Coaches and athletes routinely monitor a range of metrics with the hope of gaining insight into how an athlete responds to their training. These can include measures of training load (duration and intensity), heart rate variability (HRV), sleep, diet, and daily measures of subjective well-being, among others (Bourdon et al. 2017). Despite careful planning, there can still be large discrepancies between the training stimulus prescribed by coaches and experienced by athletes (Voet et al. 2021). Improved understanding of an athlete’s training response could allow a training plan to be better tailored to an individual’s needs, help minimize the risks of non-functional overreaching, illness, and/or injury (Halson 2014), and improve the adaptive response to training (Figueiredo et al. 2022; Nuuttila et al. 2022).

Training load refers to the combination of training volume and intensity, and can be measured and classified as either external or internal depending on whether the measurable aspects occur externally or internally to the athlete (Impellizzeri et al. 2019). External training loads are characterized by measures such as distance, power, or speed, whereas internal loads can be represented by heart rate (HR), blood lactate, and session rating of perceived exertion (sRPE) (Halson 2014). Internal load reflects the relative physiological strain from training and has been recommended as the primary measure when monitoring athletes (Impellizzeri et al. 2019). Internal load plays a pivotal role in determining training outcomes and can also reflect variations in the stress response to a given external load due to other stressors such as extreme temperature, or accumulated training fatigue (Impellizzeri et al. 2019). Coaches often use subjective wellness ratings by athletes for monitoring purposes, which may include questions about fatigue, soreness, sleep, and mood, and are sensitive to fluctuations in training load (Clemente et al. 2019a; Saw et al. 2016). These ratings reflect the athletes’ perceptions of their well-being in response to the intensity of their training. However, much of the research on the relationship between training load, sRPE, and wellness has been in team sports and not endurance sports, and has not accounted for potential interactions between training load, sleep, and dietary intake.

From a nutrition perspective, athletes and sports nutritionists are continually challenged to balance the nutritional demands of training while also optimizing body composition and promoting skeletal muscle adaptation. Increasing energy and carbohydrate intake during periods of intensified endurance training can attenuate symptoms of overreaching (Achten et al. 2004), yet many athletes routinely train in an overnight-fasted state and/or restrict carbohydrate intake before exercise (Rothschild et al. 2020). The interaction between dietary intake and training quality in the context of longer-term, self-selected training and nutrition intake has not been well characterized. Although logistically challenging, investigating longer-term dietary intake during endurance training could help elucidate the role of self-selected nutrition intake on daily recovery during endurance training. The increased availability of valid and user-friendly mobile food-tracking apps can help facilitate data collection while minimizing disruption to an athlete’s training and lifestyle.

The relationship between training, diet, sleep, and other lifestyle factors is complex, as many factors converge which may have non-linear and/or temporal relationships, with one often influencing the other. This underscores the need for more advanced tools for understanding athlete readiness and well-being. Machine learning techniques have been increasingly used in sports science, particularly in the context of multi-factorial data such as predicting injuries (Van Eetvelde et al. 2021) and subjective well-being (Perri et al. 2021), as well as in nutrition research to model complex nutrient interactions and address confounding variables (Morgenstern et al. 2021). However, to our knowledge machine learning has yet to be used to predict an endurance athlete’s perceived recovery or HRV based on a combination of factors routinely monitored by athletes and coaches. Therefore, the goal of this study was to test the ability of machine learning models to predict perceived morning (AM) recovery status and daily change in HRV based on training metrics, dietary intake, sleep, HRV, and subjective well-being. Secondary aims were to highlight the most important variables for accurate prediction, and to examine the influence of factors that can tangibly be manipulated by coaches and athletes. It is hoped that such information can allow coaches to focus on a subset of variables with the strongest predictive power.

## Methods

### Study design

This observational study monitored the daily self-selected nutrition intake, exercise training, sleep habits, HRV, and subjective well-being of endurance athletes for 12 weeks. Throughout the study period, participants were free to perform any type of exercise and consume any type of diet. Measures of diet, training, sleep, HRV, and subjective well-being were recorded daily. Models were created for two primary outcome variables—one subjective measure (AM perceived recovery status (PRS) score) and one objective measure (change in resting HRV from the previous day, HRV change). The study was open to males and females aged 18 or older who train at least 7 h/week, were using a smartphone app to track their dietary intake at least 5 days/week, capture HRV daily, and track sleep duration using a wearable device. All study protocols and materials were approved by the Auckland University of Technology Ethics Committee (22/7), and all participants provided informed consent prior to starting the study. Data collected from the same athletes relating to the influence of carbohydrate intake on daily recovery have been reported elsewhere (Rothschild et al. 2023).

### Participants

Fifty-five endurance athletes (61.8% male, aged 42.6 ± 9.1 years, training 11.6 ± 3.9 h/week) took part in the study. The primary sports represented were triathlon (67.3%), running (20.0%), cycling (10.9%), and rowing (1.8%). The self-reported competitive level included professional (2.6%), elite non-professional (qualify and compete at the international level as an age-group athlete, 34.6%), high-level amateur (qualify and compete at national championship-level events as an age group athlete, 25.6%), and amateur (enter races but do not expect to win, or train but do not compete, 37.2%) athletes.

### Assessment of self-reported exercise

All exercise was recorded in Training Peaks software (TrainingPeaks, Louisville, CO, USA). Each session was noted for modality (e.g., bike, run, swim), total time, and a rating of perceived exertion (RPE) using the Borg CR100^®^ scale, which offers additional precision compared with the CR10 scale (Clemente et al. 2019b). Participants were instructed to rate their perceived effort for the whole training session within 1 h of exercise, although RPE scores are temporally robust from minutes to days following a bout of exercise (Foster et al. 2021). Additionally, participants noted the amount of carbohydrate (in grams) consumed within the 4-h pre-exercise window.

### Assessment of self-reported dietary intake

Details of dietary assessment have been described elsewhere (Rothschild et al. 2023). Briefly, participants were instructed to maintain their typical dietary habits and record all calorie-containing food and drink consumed for the duration of the 12-week study, using the MyFitnessPal application (www.myfitnesspal.com). Due to previous habitual use, three participants used the Cronometer application (www.cronometer.com) and one participant used the Carbon application (www.joincarbon.com). Incomplete days of tracking (2.2 ± 4.6% of days per participant) were removed from the data. To aid in compliance, participants were recruited who were already regularly tracking their diet (in several cases daily for 4+ years), and so all participants entered the study with strong intrinsic motivation for habitual diet tracking.

### Assessment of resting HRV and sleep

Resting HRV was recorded daily and analyzed using the natural logarithm of the square root of the mean sum of the squared differences (Ln rMSSD) between R–R intervals (Plews et al. 2013b). For participants using Oura ring (Oura Health, Oulu, Finland) or Whoop straps (Whoop, Inc., Boston, USA), nocturnal HRV was used, whereas measurements were taken upon waking for those using the HRV4Training (www.hrv4training.com), Elite HRV (Elite HRV, Inc., Asheville, USA), or ithlete (HRV Fit Ltd. Southampton, UK) smartphone apps. High correlations have been reported between nocturnal and morning HRV measurements (Mishica et al. 2022). Nightly sleep duration was recorded using wearable devices, which included Oura ring, Whoop strap, Applewatch, Fitbit, and Garmin models. These consumer-grade devices offer adequate accuracy in detecting sleep–wake times, but not sleep staging (Chinoy et al. 2021; Miller et al. 2020; Roberts et al. 2020; Zaffaroni et al. 2019). Further details of participant devices used for HRV and sleep tracking are shown in Supplemental Fig. 1.

### Assessment of subjective well-being

Each morning, participants answered four questions related to subjective well-being, which have been shown to respond consistently to training-induced stress (Saw et al. 2016). The PRS scale (Laurent et al. 2011) was used to measure overall recovery with athletes manually typing a number into Training Peaks software. The 100-point version of the scale was used, which has been shown discriminate between answers better than the 10-point scale (Clemente et al. 2019b). In addition, ratings of life stress (1–7), sleep quality (1–7), and muscle soreness (1–10) were also recorded into the software each morning (Supplemental Fig. 2). Participants were familiarized with all scales prior to starting the study. In addition, participants were asked to record their body mass at least one time per week in the morning.

### Data preparation

Training load was calculated for each workout as the product of RPE and duration of exercise in minutes (sRPE, Haddad et al. 2017), divided by 10 to account for the 100-point scale. Exercise was summed into daily totals for workout duration and training load, along with coded variables for modality of workout (e.g., swim, bike, run, strength, other) and if any training was performed in the fasted state. Because dietary protein and fat ingestion have minimal effects on substrate oxidation (Rothschild et al. 2022), fasted training was defined as consuming < 5 g of carbohydrate in the 4-h pre-exercise window. For multiple exercise sessions in a single day, a weighted mean based on the duration of each session was used to calculate a single daily value for pre-exercise carbohydrate ingestion. External load metrics such as power or pace were not collected because many athletes undertake activities that cannot be quantified on a common scale such as strength training, yoga, or swimming. This was deemed acceptable because sRPE is considered to be a valid and reliable method for calculating training load across modalities (Haddad et al. 2017). Seven-day rolling measures for training monotony (a measure of day-to-day variability in the weekly training load, calculated as average daily load divided by the standard deviation) and training strain (product of total weekly training load and training monotony) were calculated (Haddad et al. 2017). Exponentially weighted 7-day moving averages of training load, HRV, and resting HR were calculated to account for residual effects of recent training (Plews et al. 2013a). A sleep index value was calculated as the product of sleep duration and subjective sleep quality (Sawczuk et al. 2021). Daily training volume (hours per day) and training load for each participant are presented in Supplemental Figs. 3 and 4.

Participants were excluded from the analysis if they trained an average of less than 6 h/week (*n* = 8) or did not log at least 85% of the required data points (*n* = 4). Participants who did not complete the full 12 weeks due to illness, injury, or dropping out but completed at least 6 weeks of tracking were included in the analysis (*n* = 11). Among participants included in the analysis (*n* = 43), 2.4 ± 1.7% of data points were missing. Missing values were imputed at the individual level using multiple linear regression and nearest neighbor algorithms for diet and training measures, and using median values for other variables (Kuhn and Johnson 2013).

To increase the available options for modeling and interpretation, the data were transformed from a time series into independent observations. A time series is a sequence of data points at equally spaced points in time and ordered chronologically. Time series data cannot be analyzed with common techniques such as linear modeling if the day-to-day observations are correlated with observations at previous time points (i.e., auto-correlated) and are not independent of each other, as key assumptions of linear regression are violated (James et al. 2021). To account for this, a process of Markov unfolding (Truda 2021) was used. This is based on the Markov assumption, whereby the values in any state are influenced only by the values of the immediately preceding or a small number of immediately preceding states (Gudivada et al. 2015). Data were analyzed for autocorrelation, and it was determined that a maximum of 7 previous days could have a relevant influence on a given day’s data. This makes logical sense, as many behavioral and training schedules follow a weekly cycle. The process of Markov unfolding entails copying the columns of the original dataset, shifting them down by one row, and stacking them as new columns on the right of the dataset (labeled as lag 1). This is repeated with shifts of 2 − *n*, where *n* is the number of previous days to be included. The first *n* rows from the beginning of the dataset are discarded, as there are missing values for some of the lags. This results in a dataset that is a few rows shorter, but *n* + 1 times wider than the original dataset and the observations can be treated as totally independent, allowing the use of any modeling approach that assumes independent data. This approach to making the dataset ~ 7× wider can result in the curse of dimensionality, whereby the test error tends to increase as the dimensionality of the problem (i.e., the number of predictors) increases (James et al. 2021), but this may be mitigated by the use of algorithms which use regularization to conduct feature selection (Kuhn and Johnson 2013). It should be noted that the variables created as 7-day rolling averages would allow the previous 14 days of information to be provided to the model (i.e., a 7-day average from 7 days ago). All analyses were carried out with R version 4.0.3 (the R foundation for Statistical Computing, Vienna, Austria). Descriptive statistics are provided as mean ± SD.

### Models

A series of models were built for the two outcomes of interest—AM PRS score and daily change in HRV, at both the group level (full dataset) and for each individual participant. To account for between-person differences in AM PRS scores and improve both interpretability and generalizability, all values were centered around each participant’s mean value. This results in each participant having a mean of 0, with a 1-unit change in AM PRS in relation to each participant’s average value. To reduce multicollinearity, highly correlated predictors (Pearson correlation > 0.85) were removed from the dataset prior to modeling by removing the one with the largest mean absolute correlation with the rest of the data (Kuhn and Johnson 2013). For each outcome, models were made using the primary subset of variables (MAIN) and a subset of variables that can tangibly be manipulated by athletes/coaches (ACTIONABLE). The included variables are shown in Table 1. Descriptive statistics of these variables are provided in Supplemental Table 1. At the group level, models for each outcome were made using the two variable subsets (MAIN and ACTIONABLE). For comparison with these models, two linear regression models were created—a linear mixed effects model using the five variables with the highest importance scores from the MAIN model as fixed effects and participant ID specified as a random effect (top five from group MAIN), and an intercept-only model as a baseline comparison that simply predicted the mean value. The mixed effects model was chosen to determine how a very limited subset of variables would perform, and the intercept-only baseline was used to establish a realistic upper bound for the root mean squared error (RMSE), as useful prediction models should have lower RMSE values. At the individual level, in addition to the MAIN and ACTIONABLE models, linear models were made consisting of the five variables with the highest importance from the MAIN group model (top five from group MAIN), the five variables with the highest importance from their own respective MAIN model (top five from individual MAIN), and an intercept-only model as a baseline comparison.

**Table 1:** Overview of variables included in the modeling

Category	Variables
Training	Exercise duration (min), modality, fasted training (yes/no), number of workouts per day, number of consecutive training days, session rating of perceived exertion (sRPE, highest for a single session each day and a duration-based weighted average for the day), training load (TL; min × sRPE), 7-day exponentially weighted and non-weighted moving average of TL, 7-day highest single-day TL, training monotony (weekly mean TL/weekly SD), training strain (weekly load × monotony), training feeling (TF), day of the week
Dietary	Total kcal, carbohydrate (CHO, g/kg), fat (g/kg), protein (g/kg), pre-exercise CHO (g), 3-day and 7-day moving averages of CHO, fat, protein, and kcal intake, 7-day moving average standard deviation of daily CHO intake and CHO monotony (weekly mean intake/weekly SD)
Sleep	Sleep duration (hours), *sleep index (sleep duration* × *quality),* 7-day moving average sleep duration and *sleep index*
Subjective measures	*Perceived recovery status (PRS)*, soreness, life stress, *sleep quality*
Non-exercise	*Resting HRV* and *resting HR* (daily, change from previous day, and 7-day moving averages of each)
Planned interactions	AM PRS: 7-day average TL × 3-day average CHO intake 7-day training monotony × 3-day average CHO intake HRV: prior-day TL × sleep duration Prior-day TL × prior-day AM PRS score
Subject characteristics	Participant ID, age, HRV app, sleep app, percentage of missing data, competitive level, primary sport, training age, body weight
Top five Variables from group MAIN	AM PRS: AM PRS 1 and 2 days ago, soreness, life stress, sleep quality HRV: 7-day average HRV change 1 day ago, HRV change 1, 2, and 7 days ago, HRV 1 day ago

Italics indicate variables that were removed from the ACTIONABLE models. Descriptive statistics for these variables are provided in Supplemental Table 1

Nine different learning algorithms, including parametric and non-parametric methods, were trained for each model using the *Tidymodels* ecosystem in R. These included three linear regression models with regularization (Ridge, least absolute shrinkage, and selection operator (LASSO), and LASSO with pre-specified interaction terms), three non-linear regression models (multivariate adaptive regression spline (MARS), support vector machine (SVM), and K-nearest neighbors (KNN)), two ensembles of decision trees (XGBoost and Light GBM), and a single layer neural network (NNET). The best model was chosen using leave-one-subject-out cross-validation (Kunjan et al. 2021), which means models were fit using data from all but one participant, with that participant serving as an out-of-sample test set. This form of cross-validation (e.g., 43-fold cross-validation) allows internal validation of the model while keeping a given participant out of both training and testing sets (i.e., data leakage). The performance of the developed model was then taken as the average of the performance measures (Collins et al. 2024). For all group models, the LASSO model was selected as the best-performing model.

Variable importance (a measure of the strength of the relationship between the observed values of the variable and the observed response) for the MAIN group models was determined using a permutation-based approach, which measures a feature’s importance by calculating the increase of the model’s prediction error after permuting the feature (Biecek & Burzykowski 2021). In addition to variable importance, partial dependence profiles were created to aid in model interpretation, which show how the expected value of a model prediction changes after accounting for the average effect of all other variables (Biecek and Burzykowski 2021).

The individual models used the same algorithms mentioned above. Tenfold cross-validation was repeated ten times, with the best algorithm and parameter set chosen based on the lowest RMSE. Accuracy metrics were calculated using 500 bootstrap resamples. Variable importance was calculated using a model-based approach (Greenwell et al. 2020) and scaled so the total importance summed to 1. Because individual models could use different algorithms for each participant, scaling the importance allowed a summarization of importance across different model types by taking the mean values. To compare performance among the five types of individual models (best model MAIN, best model ACTIONABLE, top five from group MAIN, top five from individual MAIN, and baseline intercept-only model), a linear mixed model was used, with RMSE from each model used as the dependent variable, model type specified as a fixed effect, and participant ID specified as a random effect. Estimated means were calculated using the *Emmeans* R package and comparisons made using the Sidak test.

## Results

**Fig. 1:**
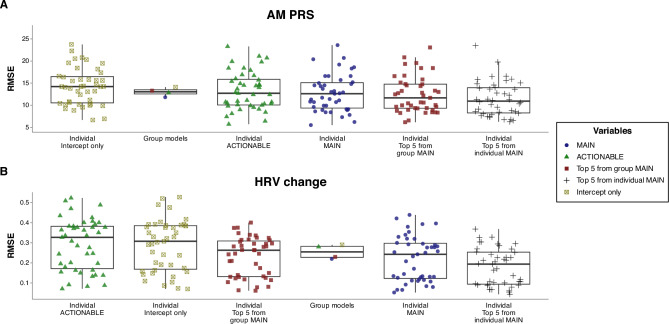
Root mean squared error (RMSE) of the group and individual models, separated by the variable set included in the model and ordered by mean RMSE values. For group models, “top five from MAIN” represents a linear mixed model with the top five features from the MAIN group model based on variable importance scores. For individual models, “top five from group MAIN” represents a linear model with the same top five features from the MAIN group model, and “top five from individual MAIN” represents a linear model with the top five features from each participant’s MAIN model. RMSE values were determined using out-of-sample data for group models and using 500 bootstrap resamples for individual models

**Fig. 2:**
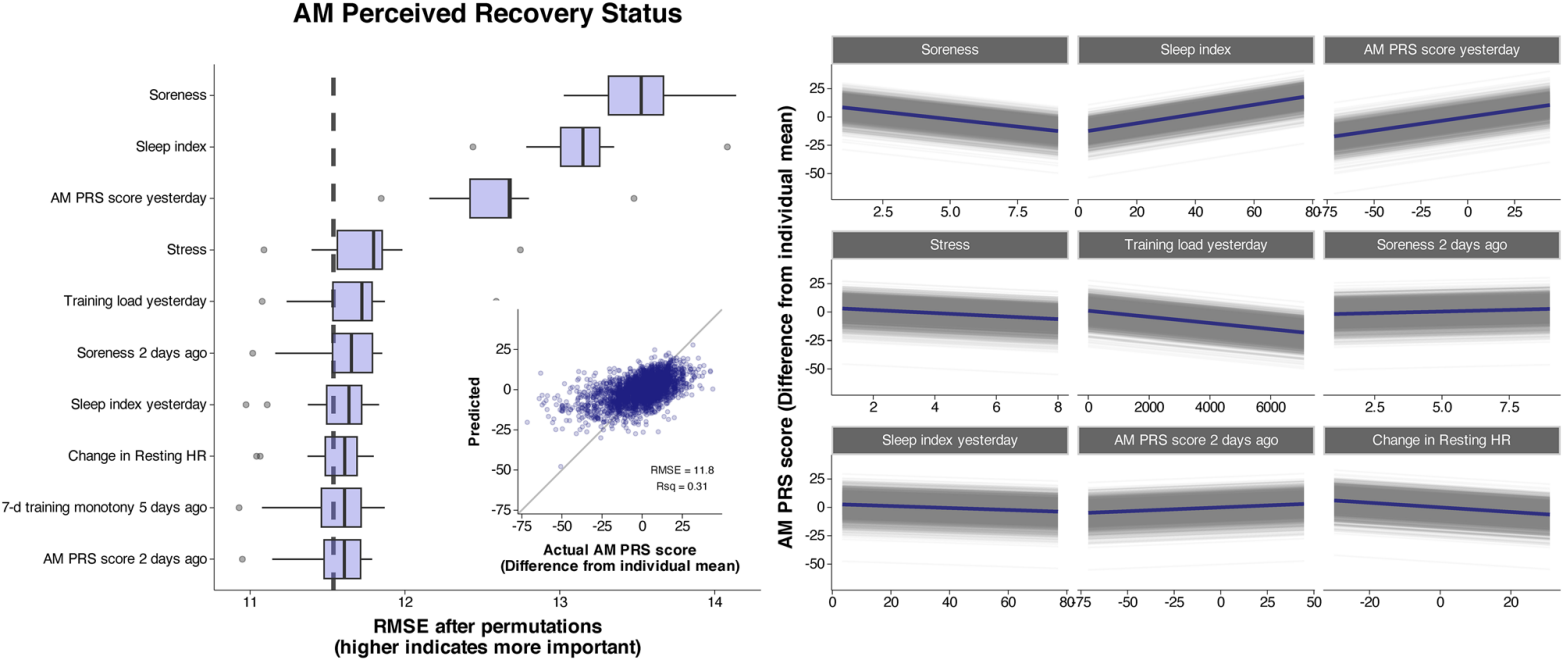
AM perceived recovery status (PRS) group model results using MAIN variables. Top ten most important variables based on permutation-based feature importance are shown in a boxplot, along with a scatterplot of actual vs. predicted values on an out-of-sample dataset (inset), and partial dependence plots for the top nine continuous variables (right), where colored lines represent the average of all observations shown individually as gray lines. The vertical dashed line in the boxplot represents the full model RMSE from the training dataset

**Fig. 3:**
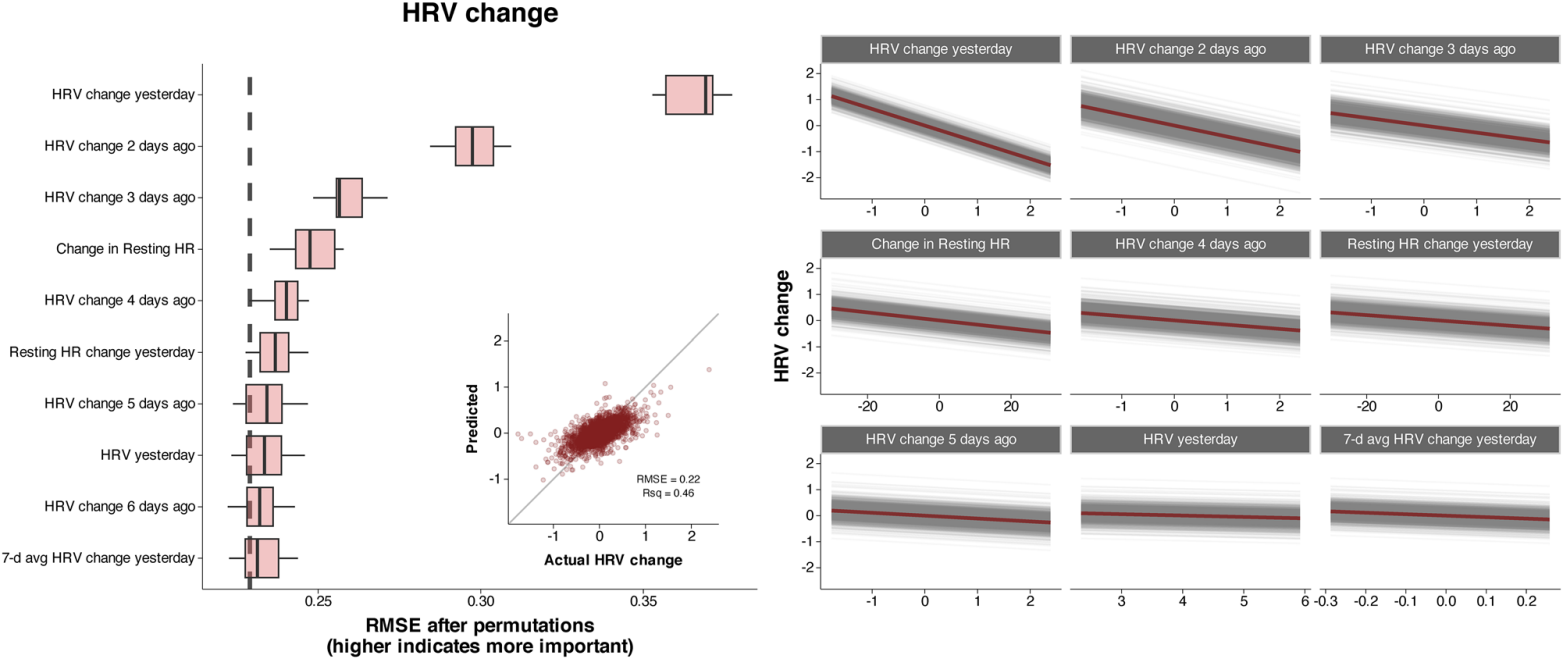
HRV change group model results from the best-performing model using MAIN variables. The top ten most important variables based on permutation-based feature importance are shown in a boxplot, along with a scatterplot of actual vs. predicted values on an out-of-sample dataset (inset), and partial dependence plots for the top nine continuous variables (right), where colored lines represent the average of all observations shown individually as the gray lines. The vertical dashed line in the boxplot represents the full model RMSE from the training dataset

**Fig. 4:**
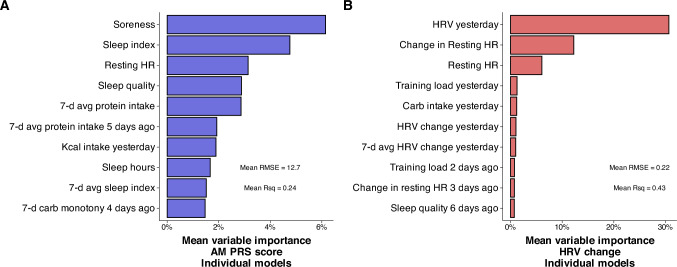
Variables with the highest mean variable importance scores from the individual MAIN models for AM perceived recovery status (PRS) (A) and HRV change (B) models. Root mean squared error (RMSE) and R-squared (Rsq) values shown are the average of the individual MAIN models

A total of 3572 days of tracking were included in the analysis (83.1 ± 9.9 per participant). Average participant training volume was 11.8 ± 3.3 h/week. Mean daily dietary intake was 39.6 ± 8.7 kcal/kg, 4.1 ± 1.5 g/kg carbohydrate, 1.9 ± 0.4 g/kg protein, and 1.7 ± 0.5 g/kg fat. Average sleep duration was 7.5 ± 0.7 h per night. Values for the main outcome variables were 0.0 ± 14.3 and 0.0 ± 0.31 for AM PRS and HRV change, respectively. Density plots showing the distribution of the two main outcome variables for each participant are shown in Supplemental Fig. 5. MAIN group models demonstrated improved accuracy compared with the baseline model (Table 2). The accuracy of the individual models was improved compared with the baseline models (Table 3), but varied more than fivefold across participants (Fig. 1). Figures 2 and 3 show the ten variables with the highest importance from the group modeling for AM PRS (Fig. 2) and HRV change (Fig. 3), as well as a scatterplot comparing predicted vs. actual values (inset into each figure) and partial dependence plots showing how the expected value of a model prediction changes based on these variables. Figure 4 shows the ten variables with the highest mean importance scores across all participants for the individual MAIN models.

**Table 2:** Accuracy of group models

Outcome	Model	Variables	RMSE [95% CI]	*R*^2^
AM PRS	LASSO	MAIN	11.8 [10.7, 12.9]	0.31 [0.26, 0.34]
AM PRS	LASSO	ACTIONABLE	12.8 [11.6, 14.0]	0.20 [0.16, 0.24]
AM PRS	LMM	Top five from MAIN	13.3 [12.2, 14.4]	0.29 [ 0.25, 0.33]
AM PRS	Baseline	Intercept only	14.1 [12.8, 15.3]	0
HRV change	LASSO	MAIN	0.22 [0.19, 0.25]	0.46 [0.22, 0.51]
HRV change	LMM	Top five from MAIN	0.23 [0.20, 0.26]	0.35 [0.32, 0.37]
HRV change	LASSO	ACTIONABLE	0.28 [0.24, 0.32]	0
HRV change	Baseline	Intercept only	0.29 [0.25, 0.32]	0

*AM PRS* AM perceived recovery status, *LASSO* linear regression model with regularization, *LMM* linear mixed model with participant ID specified as a random effect, *RMSE* root mean squared error, in units of the original measurement (0–100 for AM PRS and Ln rMSSD for HRV change)

**Table 3:** Accuracy of individual models

Outcome	Model	Variables	RMSE^a^	*R*^2^
AM PRS	Linear	Top five from individual MAIN	12.0 ± 5.2^a^	0.35 ± 0.20
AM PRS	Linear	Top five from group MAIN	12.4 ± 4.1^ab^	0.29 ± 0.16
AM PRS	*	MAIN	12.7 ± 4.2^ab^	0.24 ± 0.14
AM PRS	*	ACTIONABLE	13.2 ± 4.3^b^	0.20 ± 0.12
AM PRS	Baseline	Intercept only	14.2 ± 4.2^c^	0
HRV change	Linear	Top five from individual MAIN	0.18 ± 0.09^a^	0.62 ± 0.14
HRV change	*	MAIN	0.22 ± 0.11^b^	0.43 ± 0.18
HRV change	Linear	Top five from group MAIN	0.23 ± 0.10^b^	0.37 ± 0.10
HRV change	Baseline	Intercept only	0.29 ± 0.13^c^	0
HRV change	*	ACTIONABLE	0.29 ± 0.13^c^	0.10 ± 0.10

*AM PRS* AM perceived recovery status, *RMSE* root mean squared error, in units of the original measurement (0–100 for AM PRS and Ln rMSSD for HRV change)

*For MAIN and ACTIONABLE models, values are from the single best-performing algorithm (LASSO: 30%, SVM: 30%, XGBoost: 23%, Light GBM: 12%, KNN: 4%, Ridge: 1%, and MARS: 0.4% of models). All metrics were established using 500 bootstrap resamples

^a^Within each outcome, models not sharing any letter are significantly different by the Tukey test at the 5% level of significance

## Discussion

Athlete monitoring can help coaches better understand how an athlete is adapting to a training program, minimize the risk of developing non-functional overreaching, illness, and/or injury (Halson 2014), and improve training outcomes (Figueiredo et al. 2022; Nuuttila et al. 2022). This study utilized a novel approach to monitoring endurance athletes throughout 12 weeks of self-selected training to better understand the factors that can predict an athlete’s day-to-day recovery and well-being. Key findings from this study are (1) day-to-day recovery measures can be predicted based on commonly measured variables, (2) a small subset of variables offers similar predictive capability as the full dataset, (3) predictive accuracy varies greatly at the individual level, and (4) remote monitoring of multiple training, diet, sleep, and recovery measures can be performed throughout longer-term training in real-world environments.

### Model performance

All models constructed using the MAIN variables outperformed the baseline model, demonstrating utility of the tracked variables. Unexpectedly, performance at the group level of the ACTIONABLE models for HRV was poor, indicating they alone do not offer any added value for predicting the daily change in HRV. As shown in the scatterplots in Figs. 2 and 3, prediction accuracy was generally worse for scores on the upper and lower ends, likely due to the small number of extremely low or high values with which to train the models. At the individual level, the most striking finding was the large degree of variation in model performance (Fig. 1). This suggests key variables may be missing from the models that could disproportionately affect some athletes more than others. For example, alcohol intake, acute illness, and the menstrual cycle are known to influence HRV (Altini and Plews 2021) and were not tracked in this study. Moreover, participants spent only ~ 10% of their waking hours engaged in exercise, indicating the potential for many non-exercise factors to influence recovery and wellness such as walking, job-related physical activity/stress, massage, sauna, and/or ice baths. Future studies could expand on the current work by accounting for some or all of these additional factors.

At the group level, the LASSO regression models displayed the best overall performance. The model is well suited to handle a large number of predictors because it uses regularization to reduce the estimated coefficients toward zero (James et al. 2021). Put simply, this type of model uses built-in feature selection to remove non-needed variables from the model. We also used a linear mixed effects model of the top five variables based on variable importance scores, with participant ID included as a random effect. Both the LASSO and linear mixed models performed well (Table 2), suggesting they both can be value tools and that more complex machine learning algorithms (e.g., XGBoost or Neural Networks) may not be needed in this context.

When constructing individual-level MAIN and ACTIONABLE models, the single best model from the suite of machine learning algorithms was chosen as the accepted model. Two linear regression models were then made, using the top five variables from the group and individual MAIN models. The best performance was achieved from the linear models with the top five individual variables (Table 3), highlighting the importance of a very small subset of variables that coaches and practitioners could pay closer attention to. Importantly, the difference in performance between linear models of the top five from individual and top five from group models highlights the fact that the most important variables for each athlete will be different (Table 3). From a practical perspective, a prudent approach might be to start by monitoring a wide array of variables and reduce the number based on feedback from initial models.

### Variable importance

The variable importance calculations of the group-level models revealed a small number of variables having a disproportionately large influence on prediction accuracy (Figs. 2 and 3). This finding is corroborated by the generally good performance of the linear mixed models, which included only the top five variables, and implies the ability for coaches and practitioners to focus on just a few of the many variables that are routinely monitored. These include muscle soreness, life stress, and sleep quality for AM PRS scores, and the changes in HRV over recent days for predicting the current day’s change in HRV. However, when trying to predict at the individual level, the chosen variables should be specific to the individual. The aggregated importance scores from the individual models shown in Fig. 4 are far more diverse than at the group level, supporting the notion that the most important variables vary among different athletes. For example, among two participants with the lowest RMSE values for the AM PRS models, the most important variables were muscle soreness, prior-day PRS scores, and prior-day protein intake for one athlete, while the top variables for another athlete were all related to sleep (prior night, previous nights, and 7-day rolling averages). This suggests coaches can narrow down the focus to a few key factors that have an outsized impact on how the athlete feels. The importance of the individual differences is further evidenced by the improved performance (2–17% improvement in RMSE) of the individual linear models that used the individual’s top five variables compared with the top five group variables (Table 3).

### Explain vs. predict

The priority of a statistical model can be to explain (i.e., test causal explanations), predict (new or future observations), or describe the data structure in a compact manner (Shmueli 2010). The focus of this analysis was on predictive power, for several reasons. The observational nature of our data from free-living environments is better suited to predictive modeling, whereas laboratory-controlled experimental data are better for explanatory modeling (Shmueli 2010). In the context of a large dataset with complex relationships, predictive modeling can help uncover potential new causal mechanisms and lead to the generation of new hypotheses (Shmueli 2010). This is reflected in the variable importance scores, particularly for HRV change, where few of the top predictors could be thought of as having any causal role. However, new hypotheses could be generated relating to a potential reversion to the mean effect for HRV, for example, based on the negative relationship between the top predictors and the daily change in HRV (Fig. 3, partial dependence plot). From a practical perspective, use of these models should be limited to communicating the expected values for an athlete on a given day, rather than suggesting ways to modulate the variables of interest. Future studies could elucidate the role of perceived recovery status and HRV on training quality, longer-term training adaptations, and overtraining syndrome, as well as investigate causal relationships between key variables from this study and training outcomes.

### Athlete monitoring

Direct monitoring of training and fatigue responses is common in high-performance sport environments (Bourdon et al. 2017). Better understanding of an athlete’s response to training and recovery could help coaches improve the effectiveness of a training program. However, it is challenging to control for, or even account for, the large number of variables potentially influencing an athlete’s response to training, particularly over longer time frames. Observational studies can help to answer questions that would not be feasible to study in a controlled laboratory environment. A strength of this study design is the length of monitoring period, which allowed athletes to capture a range of daily and weekly training volumes. Advances in technology have also opened far more opportunities to gather valid and reliable data from athletes in their home training environments (Kinnunen et al. 2020; Plews et al. 2017). Although dietary intake can often be underreported, nearly all previous studies have used short-duration food records rather than smartphone apps. It has been suggested that familiarity with and interest in keeping food records may lead to more reliable estimates of energy intake (Champagne et al. 2002), and in our study all participants were already habitually recording dietary intake using a smartphone app. Although this approach to gathering data would not suit all athletes, many are accustomed to daily tracking of a wide range of data, and it is likely that a model-based analytical approach could offer valuable insight.

### Machine learning

Machine learning has been increasingly used in sports science, often for predicting injuries (Van Eetvelde et al. 2021), but also for predicting subjective well-being in athletes (Perri et al. 2021). Machine learning algorithms can be criticized for their lack of transparency, but regression models with built-in feature selection (e.g., the LASSO model used in this study) offer interpretable models that can also handle high-dimensional data. Our findings are echoed by a systematic review showing no performance benefit of complex machine learning algorithms over logistic regression for clinical prediction models (Christodoulou et al. 2019). However, in our study, the machine learning models played a critical role in being used to identify the top variables for the linear models.

### Limitations

Limitations of this study relate to the observational and uncontrolled nature of the data collection, the large number of variables collected, and the potential for important factors to have not been collected such as alcohol intake and the menstrual cycle. Participants were required to record their training, diet, sleep, HRV, and subjective well-being daily for 12 weeks. We specifically recruited people who were already doing this routinely, as this approach would not be practical for all athletes. Data integrity was checked based on the number of missing values, and by looking for trends in dietary reporting that could not be explained by changes in training load or body weight. Nonetheless, it is possible that participants did not always enter data as accurately as possible. There is also the risk of bias in reporting if an athlete is aware that their coach or a researcher will be seeing their data, answering based on what they think is desirable. Despite capturing a wide range of variables, we only had a single measure of internal training load and no measure of external load. This was done to accommodate athletes training across a variety of endurance and strength training modalities. Future research in single-sport athletes (e.g., cyclists or runners) would allow additional load metrics like HR, total work, or distance to be more easily factored into the modeling. In addition, energy availability, alcohol intake, and menstrual cycle tracking would be desirable metrics to include. Future work could also benefit from using continuous sliding scales for subjective well-being measures that would allow decimal places to be recorded, rather than the 7- or 10-point integer scales built in to the training monitoring software. This was the reason we used the 100-point, rather than 10-point PRS scale (Clemente et al. 2019b). Finally, no performance measures were captured, leaving the ultimate utility of this approach unclear.

## Perspective

To our knowledge, this is the first study of its kind to track this diverse range of self-selected and self-reported training of endurance athletes. Findings from this study, and the approach used, can enable coaches and athletes to better understand and focus on the few key measures which can offer an outsized amount of predictive capability. Although the prediction accuracy could likely be improved by capturing additional variables of interest, the current predictions offer information that is practically relevant. For example, an RMSE value of 12 from our model using the 100-point scale would translate to an average error of 1.2 when using a 10-point well-being scale, providing a coach with a useful gauge of an athlete’s readiness. These data also reveal the importance of looking into factors affecting each athlete, rather than applying group-level findings to the individual. Importantly, use of these models should be limited to communicating the expected values for an athlete on a given day, rather than suggesting ways to modulate the variables of interest. This approach can also be combined with domain knowledge to individualize key metrics for athlete monitoring and evaluation.

## Supplementary Information

Below is the link to the electronic supplementary material.

Supplementary file1 (DOCX 4915 KB)

## Data Availability

The authors are willing to discuss data sharing under collaborative agreements. Please contact the corresponding author.

## Abbreviations

HR: Heart rate
HRV: Heart rate variability
KNN: K-nearest neighbors
LASSO: Least absolute shrinkage and selection operator
MARS: Multivariate adaptive regression spline
NNET: Neural network
PRS: Perceived recovery status
RMSE: Root mean squared error
sRPE: Session rating of perceived exertion
SVM: Support vector machine

## Code availability

The R code used in this analysis is publicly available, using a synthetic dataset mimicking the original data at https://github.com/Jeffrothschild/ML_predictions_code.
