# Supplementary material for: Predicting daily recovery during long-term endurance training using machine learning analysis
Source: Eur J Appl Physiol. 2024 Jun 20;124(11):3279–90. doi: 10.1007/s00421-024-05530-2 (PMC11519101; doi:10.1007/s00421-024-05530-2)
Supplement: Supplementary file 1 — Supplementary file1 (DOCX 4915 KB) [file 421_2024_5530_MOESM1_ESM.docx]

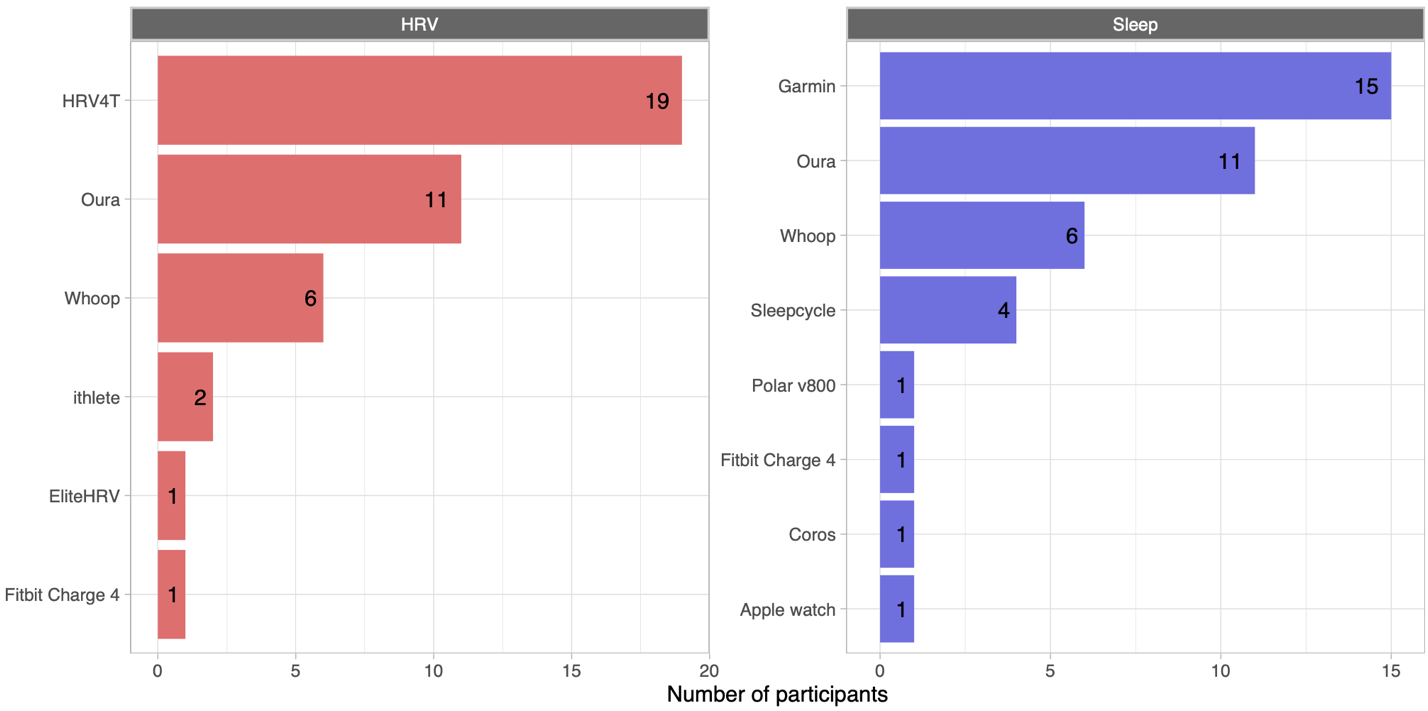


**Supplemental figure 1 -** Participant devices used for sleep and HRV tracking.


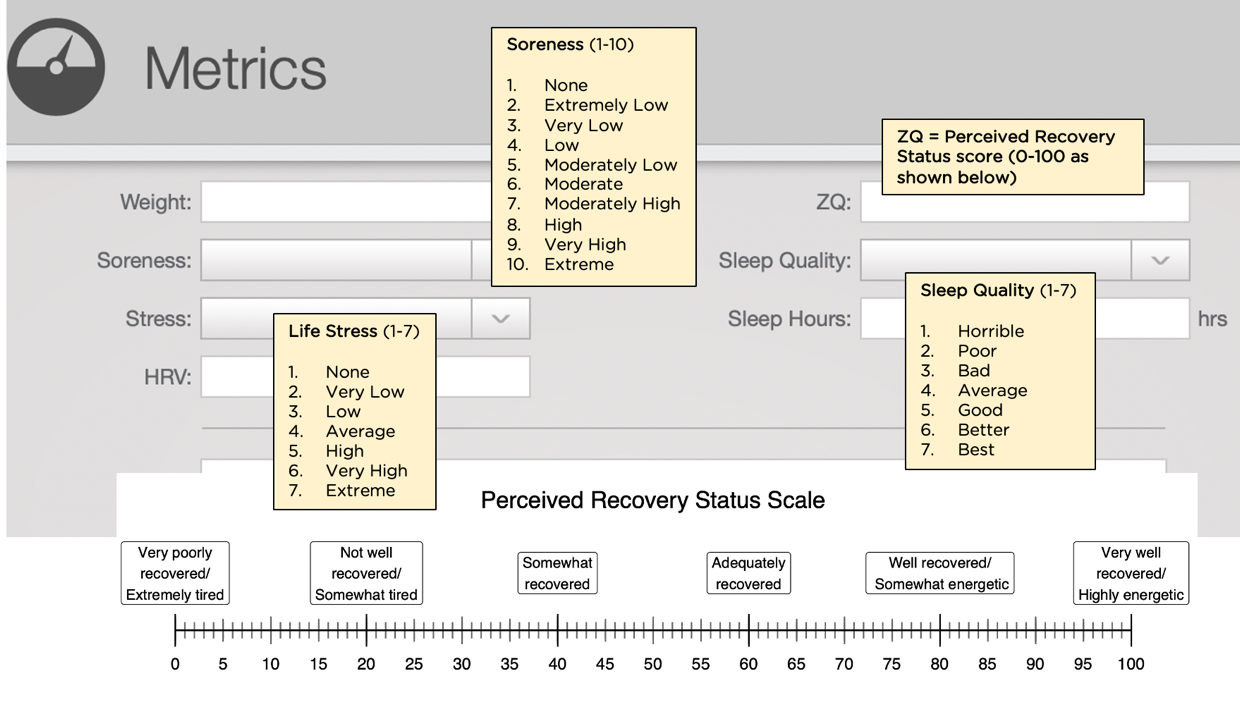


**Supplemental figure 2** – 100-pt PRS scale and scale for other subjective measures


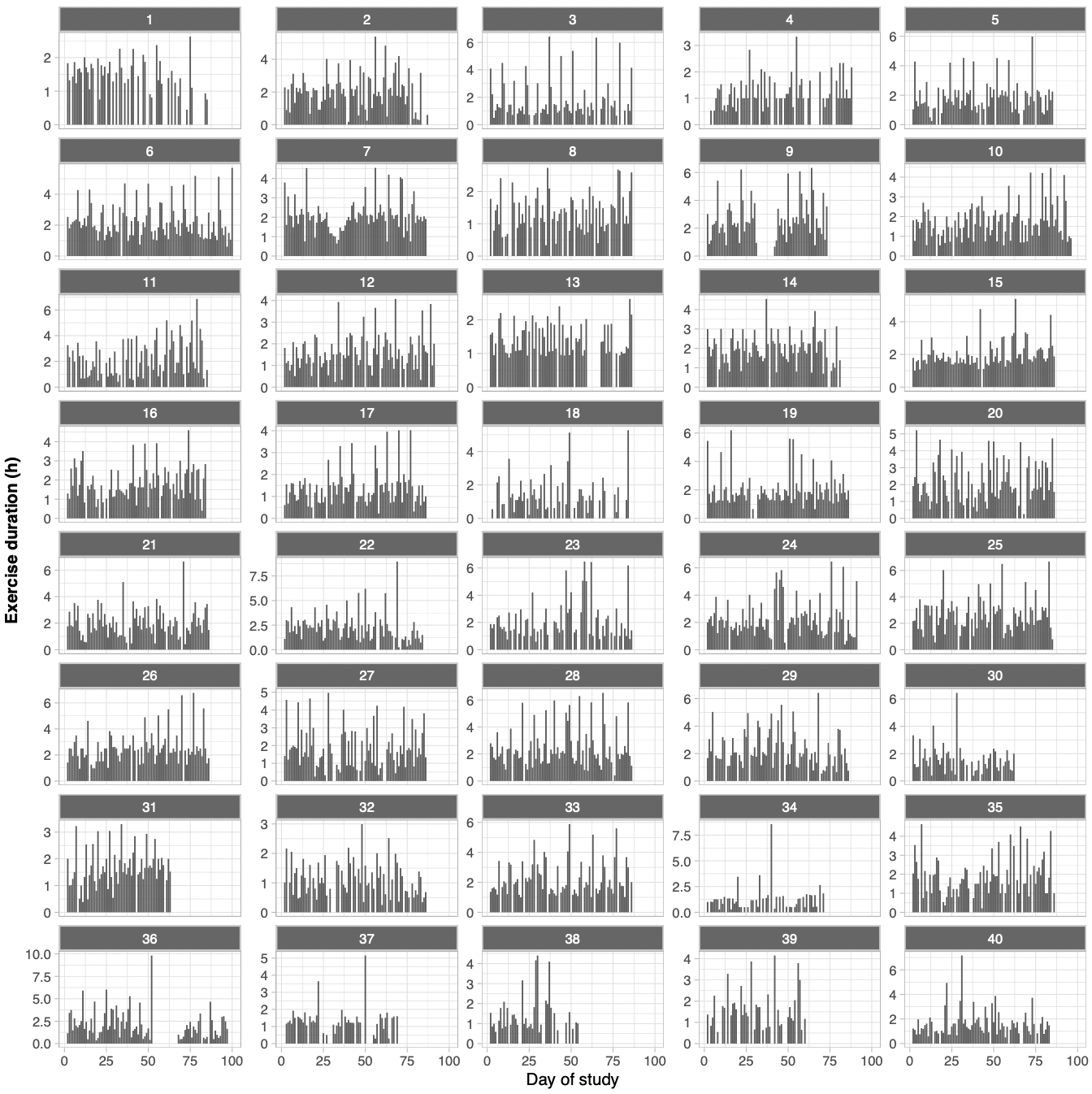


**Supplemental figure 3** – Daily training volume (hours per day) for each participant for each day of the study.


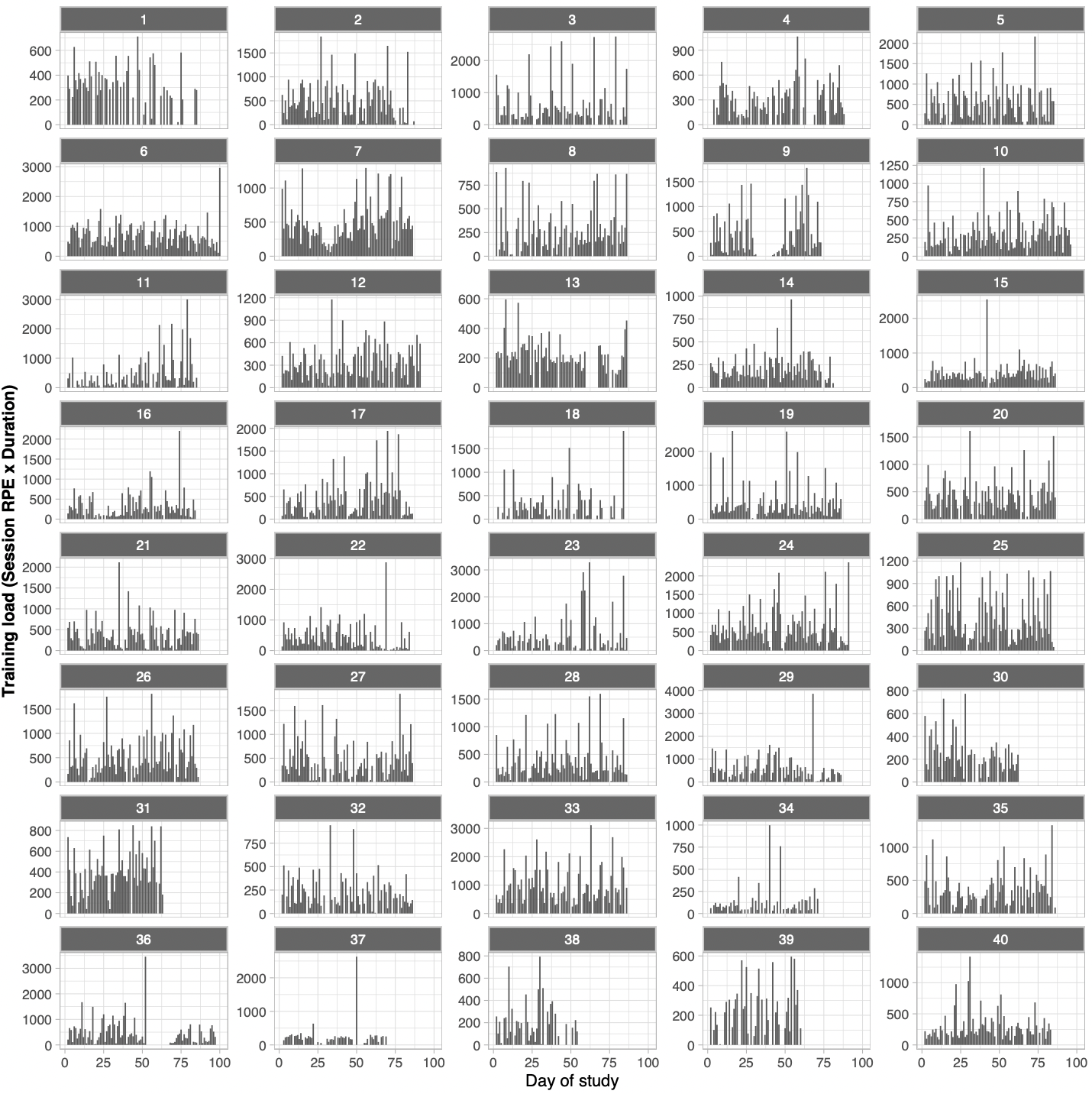


**Supplemental figure 4** – Daily training load (product of session RPE and exercise duration in minutes) for each participant for each day of the study.


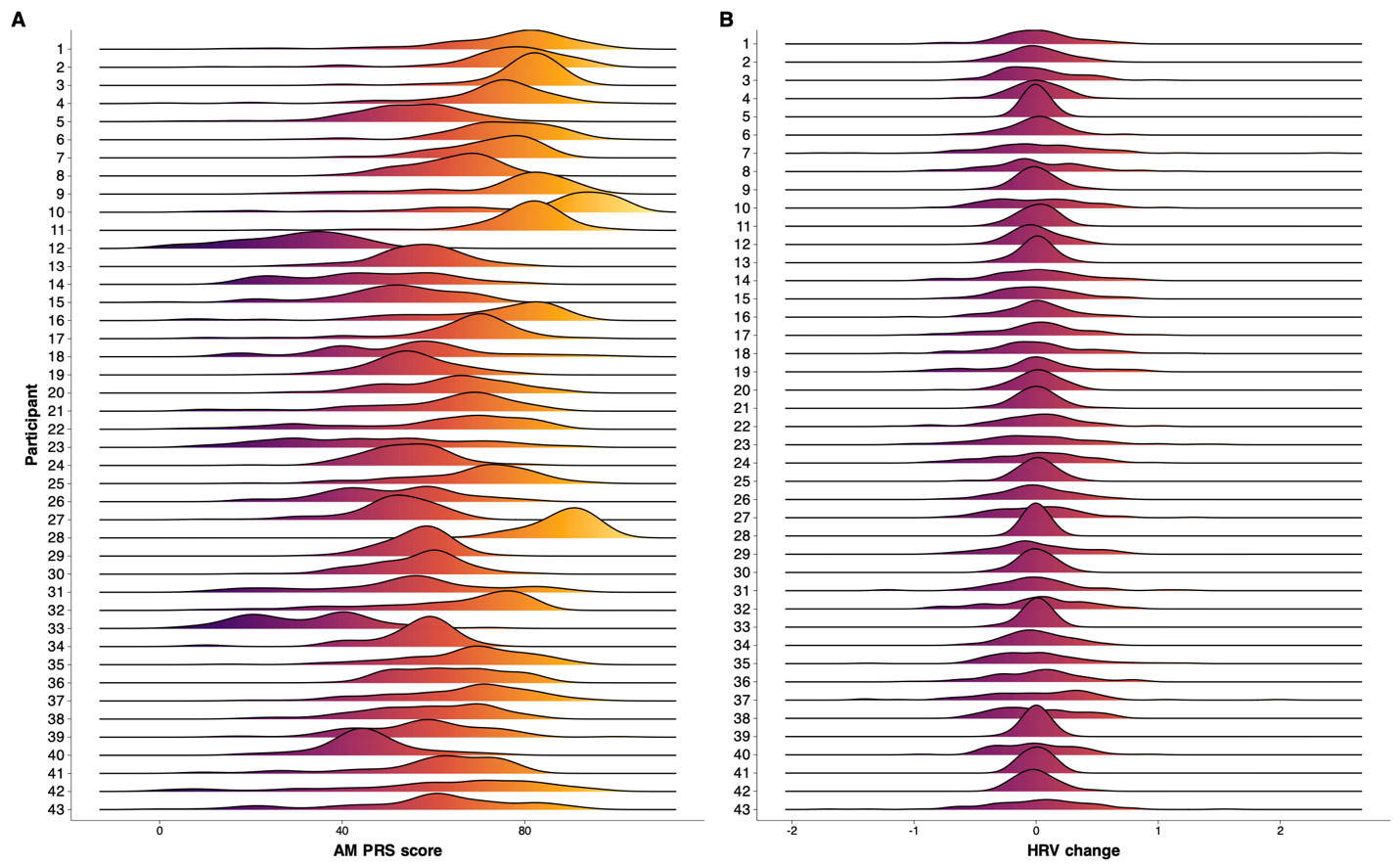


**Supplemental figure 5** – Density plot showing the distribution of the two main outcome variables for each participant. In (A), values for AM Perceived Recovery Status (PRS) are raw values, not centered.
